# *SORL1* mutations are associated with parkinsonian and psychiatric features in Alzheimer disease: Case reports

**DOI:** 10.1097/MD.0000000000025585

**Published:** 2021-04-23

**Authors:** Guozhen Qiu, Chunyan Xu, Qiwen Guo, Fei-Qi Zhu

**Affiliations:** The Third Affiliated Hospital of Shenzhen University, Cognitive Impairment Ward of Neurology Department, Shenzhen, Guangdong Province, China.

**Keywords:** Alzheimer disease, GDNF, parkinsonism, psychiatric symptoms, *SORL1*

## Abstract

**Rationale:**

The sortilin-related receptor 1 gene (*SORL1*) encodes a key protein (SORLA) involved in the pathophysiology of Alzheimer disease (AD). SORLA also mediates a trophic pathway that acts through glial cell line-derived neurotrophic factor (GDNF), a critical survival factor for the midbrain dopaminergic (DA) neurons.

**Patient concerns:**

Four patients presented to our hospital with complaints of progressive memory decline, who developed extrapyramidal signs (EPS) and psychiatric symptoms.

**Diagnoses:**

All 4 patients were diagnosed with AD based on their symptoms, findings from cranial magnetic resonance imaging, and cerebrospinal fluid analysis.

**Interventions:**

We also performed whole-exome sequencing (WES) and found 4 novel mutations in *SORL1*. Donepezil, rivastigmine, memantine, madopar, quetiapine, and risperidone were administrated as therapy.

**Outcomes:**

The four mutations would change the thermal stability of SORLA domain. This could be associated with parkinsonian and psychiatric features in AD. These patients showed improvements in parkinsonian and psychiatric features.

**Lessons:**

These cases suggest that *SORL1* mutations might result in aggregation of a-synuclein through altered function of GDNF and further lead to appearance of core dementia with Lewy bodies features.

## 1 Introduction

Alzheimer disease (AD) is a neurodegenerative disease characterized by cognitive and memory dysfunction.^[1]^ Parkinsonian and psychiatric symptoms are also common in AD patients, including bradykinesia, resting tremor, rigidity, delusions, and hallucinations.^[2,3]^ Concomitant parkinsonian and psychiatric symptoms result in a worse prognosis,^[4,5]^ however, its pathomechanism is poorly understood. The sortilin-related receptor 1 *SORL1* encodes SORLA, a key protein involved in amyloid precursor protein (APP) sorting and subsequently in degradation of amyloid-beta (Aβ) peptide, which aggregates and triggers AD pathophysiology.^[6]^ In this study, we performed whole-exome sequencing (WES) to identify novel *SORL1* mutations in 4 AD patients with parkinsonian and psychiatric features.

## 2 Methods

The study was approved by the ethics committee of the Third Affiliated Hospital of Shenzhen University. The written informed consent was obtained from each participant. The diagnosis of AD was made according to the 2014 criteria of the National Institute of Neurological and Communicative Disorders and Stroke Alzheimer's Disease and Related Disorders Association.^[7]^ Neuropsychiatric Inventory was used to evaluate psychiatric symptoms. Brain magnetic resonance imaging (MRI) was performed on a 1.5 T MRI scanner (Sonata, Siemens Medical Systems, Germany). The sequences assessed were T2W-flair axial with 5 mm thickness. Cerebrospinal fluid (CSF) was collected by lumbar puncture into polypropylene tubes. Total tau (t-Tau), phosphorylated tau (p-Tau), and Aβ42 were measured using enzyme-linked immunosorbent assays (ELISAs) (Fujirebio Inc., Tokyo, Japan). Whole-exome capture was performed using the IDT xGen Exome Research Panel v1.0 (Integrated DNA Technologies, USA) and high-throughput sequencing by the Illumina Novaseq 6000 platform (Illumina, USA). The single-nucleotide polymorphisms (SNPs) were identified by using GATK software (Genome Analysis ToolKit) (www.broadinstitute.org/gatk). Variants were annotated using ANNOVAR (http://annovar.openbioinformatics.org/en/latest/). The thermal stabilities of protein structures for the missense mutations were predicted by DUET.^[8]^

## 3 Case 1

**Figure 1:**
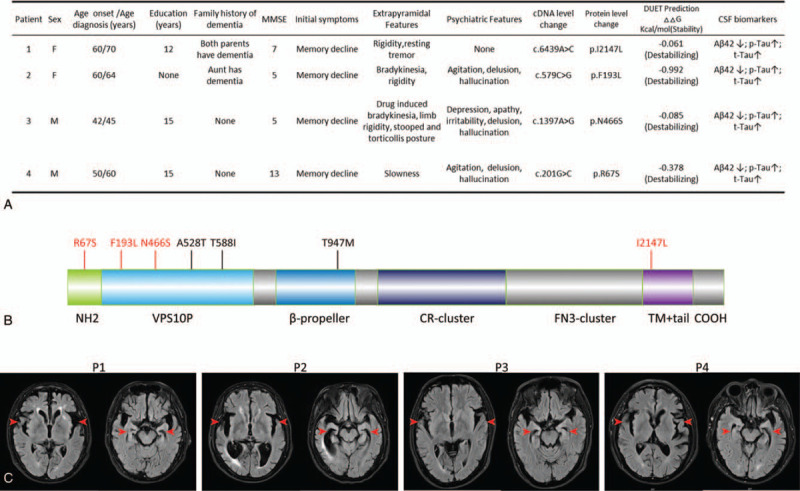
Clinical, genetic, and radiologic findings of the 4 patients. (A) Clinical and genetic findings of AD patients with *SORL1* mutations. (B) Summary of the SORLA protein structure and location of mutations. (C) FLAIR axial MRI scans showing bilateral temporal and hippocampal atrophy. AD = Alzheimer disease, DUET = a server for predicting effects of mutations on protein stability, FLAIR = fluid-attenuated inversion-recovery, MMSE = Mini-Mental State Examination, MRI = magnetic resonance imaging.

Patient 1 was a female manager with 12 years of education who, at age 60, underwent cervical cancer surgery and radiotherapy and developed gradual memory decline. She had difficulty recalling recent events, would repetitively ask the same question, and would complain of a reduced sense of smell to her daughter. After 5 years, she showed mild rigidity on her left side with resting tremor. Another 3 years later, the death of her husband worsened her memory decline. She subsequently developed disorientation, ideomotor apraxia and anomic aphasia. Physical examination showed hypertonia of the left upper limb. MRI of the brain showed bilateral temporal and hippocampal atrophy. Based on the clinical manifestations, the brain MRI findings, and CSF levels of Aβ42, P-tau, and T-tau, the patient was diagnosed as having AD with parkinsonian features (Fig. 1A, 1C). WES analysis identified a novel missense variant of *SORL1* as c.6439A>C (p.I2147L), located in the transmembrane region (Fig. 1B). DUET results demonstrated that the missense mutation destabilized the transmembrane region region of SORLA. The patient was treated with donepezil, memantine, pramipexole, and madopar, and the symptom of resting tremor was relieved after 7 days.

## 4 Case 2

Patient 2 was a housewife with a low educational level (illiterate), who presented with progressive memory loss at the age of 60, followed by a reduction in her facial expression. She regularly forgot to turn off electrical equipment and could not find her way to a new place. After 2 years, she presented with bradykinesia, limb rigidity, agitation, delusions of persecution, and visual hallucinations. Another year later, she displayed impaired language and time orientation. Physical examination revealed masked face and hypertonia of extremities. Brain MRI revealed bilateral temporal and hippocampal atrophy. Considering the clinical manifestations, the results of the brain MRI, and CSF levels of Aβ42, P-tau, and T-tau, the patient was diagnosed with AD, with parkinsonian and psychiatric features (Fig. 1A, 1C). Additionally, WES analysis identified a novel missense variant, c.579C>G (p.F193L) of *SORL1*, which was located in the vacuolar protein sorting 10 protein (VPS10p) domain (Fig. 1B). DUET results demonstrated that the missense mutation was destabilizing the VPS10p domain of SORLA. The patient was treated with donepezil, memantine, quetiapine and madopar, and she showed improvements in the psychiatric features, but no improvement in parkinsonian features in 3 months.

## 5 Case 3

Patient 3 was a male unskilled worker with 15 years of education, who developed progressive memory decline at age of 42. He would frequently forgot appointments with friends and exhibited disorientation, with deficits in language and attention. Two years later, he developed depression, apathy, irritability, delusions, and hallucinations. His wife reported that he exhibited erectile dysfunction at the age of 38. After taking Olanzapine, he developed bradykinesia, limb rigidity, and stooped and torticollis posture. His symptoms were relieved following olanzapine withdrawal. However, a follow-up examination a month later revealed a recurrence of parkinsonian symptoms. Physical examination showed generalized bradykinesia, arm rigidity, stooped posture, and slow ambulation. The patient had bilateral temporal and hippocampal atrophy in MRI of the brain. Based on the clinical features, the results of the brain MRI, and Aβ42, P-tau, and T-tau levels in the CSF, the patient was diagnosed with AD with parkinsonian and psychiatric features (Fig. 1A, 1C). A novel missense variant at c.1397A>G (p.N466S) of *SORL1*, which was located in the VPS10p domain (Fig. 1B) was identified by WES analysis. Furthermore, DUET results demonstrated that the missense mutation was destabilizing the VPS10p domain of SORLA. The patient was treated with memantine, rivastigmine transdermal patch, risperidone, and madopar, which improved his psychiatric features, without improvement in the parkinsonian features in 3 months.

## 6 Case 4

Patient 4 was a male engineer with 15 years of education who had developed progressive cognitive impairment at the age of 50. He would often forget where he parked his car and experienced disorientation while driving. Four years later, he exhibited difficulties in words-finding and calculation. He started making slow movement and felt that his left hand became rigid, he attempted not to use his left hand afterwards. He subsequently developed agitation, persecutory delusions, visual hallucinations, and difficulties in facial recognition. He also suffered from long-term constipation since the age of 45. Physical examination showed hypertonia of left limbs. Brain MRI indicated bilateral atrophy in the temporal and hippocampal regions. Based on the clinical manifestations, the results of brain MRI, and levels of Aβ42, P-tau, and T-tau in the CSF, the patient was diagnosed with AD with parkinsonian and psychiatric features (Fig. 1A, 1C). WES analysis identified a novel missense variant of c.201G>C (p.R67S) of *SORL1*, which was located in the N-terminal domain (Fig. 1B). DUET results indicated that the missense mutation destabilized the N-terminal domain of SORLA. Donepezil, memantine, and rivastigmine transdermal patch were used to treat the patient and he showed improvements in psychiatric features and slow movement in 1 month.

## 7 Discussion

We report 4 patients with AD who are carriers of 4 novel *SORL1* missense mutations. They all showed typical clinical and imaging manifestations of AD. In addition, they developed extrapyramidal signs (EPS) and psychiatric symptoms. A previous study showed that the *SORL1* variants p.T588I, p.A528T, and p.T947 M were found in AD patients who display parkinsonian features.^[9]^ Then Maple et al found that the common *SORL1* variant was associated with increased risk of dementia in Parkinson disease.^[10]^ These studies indicated that *SORL1* mutations could influence the occurrence or progression of both cognitive impairment and parkinsonian symptoms.

SORLA is involved in the APP pathway, it also mediates another trophic pathway that acts through the glial cell line-derived neurotrophic factor (GDNF), a critical survival factor for the midbrain dopaminergic (DA) neurons.^[11]^ The absence of SORLA would altered GDNF activity and lead to abnormal functioning of the DA system.^[12]^ Furthermore, it has been shown that GDNF acts against the formation of misfolded α-synuclein aggregates in DA neurons.^[13]^ An early study revealed the likelihood of parkinsonian and psychiatric features to be directly related to α-synuclein pathology in Lewy body disorders.^[14]^ We suggest that destabilizing effect of mutations on SORLA could result in the aggregation of α-synuclein through altered GDNF activity, and this can eventually lead to manifestation of core dementia with Lewy bodies features. However, several potential GDNF-binding sites of SORLA that may be harboring missense mutations, remain to be explored. In addition, although several studies have reported the co-existence of Aβ and α-synuclein,^[15]^ the underlying mechanisms are still unknown. Perhaps our observations and notion provide an alternative theory.

In conclusion, our findings expand the phenotypic spectrum of *SORL1*-related AD and offer an insight into the molecular mechanisms leading to AD with parkinsonian and psychiatric features. The limitation of this report is a lack of functional assays. Few cases have been reported so far, and further studies are needed to confirm our observations. However, *SORL1* mutations should be considered in the setting of AD with parkinsonian and psychiatric symptoms. These results could be useful for genetic counseling and future potential genotype-phenotype correlations.

## Acknowledgments

We thank the patients and their family members for cooperation.

## Author contributions

**Conceptualization:** Guozhen Qiu.

**Funding acquisition:** Guozhen Qiu.

**Investigation:** Chunyan Xu, Qiwen Guo.

**Writing – original draft:** Guozhen Qiu.

**Writing – review & editing:** Fei-Qi Zhu.
